# Preoperative aspirin continuation versus replacement therapy with low molecular weight heparin before coronary surgery: effects on postoperative bleeding risk

**DOI:** 10.1186/1749-8090-10-S1-A339

**Published:** 2015-12-16

**Authors:** Cristiano Spadaccio, Antonio Nenna, Filippo Prestipino, Gwyn W Beattie, Francesco Nappi, Fraser W Sutherland, Massimo Chello

**Affiliations:** 1 Department of Cardiothoracic Surgery, Golden Jubilee National Hospital, Glasgow, G814DY, UK; 2 Department of Cardiac Surgery, University Campus Bio-Medico of Rome, Rome, Italy; 3 Cardiac Surgery, Centre Cardiologique du Nord de Saint-Denis, Paris, France

## Background/Introduction

Management of preoperative antiplatelet therapy in coronary surgery is still variable among surgeons. Guidelines collide with prejudices: replacement of aspirin with low molecular weight heparin (LMWH) is still performed in many Centers due to a presumed minor risk of intraoperative bleeding, even though supporting evidences are weak and detrimental effects are well-described.

## Aims/Objectives

The purpose of this study is to analyse postoperative bleedings in patients scheduled for elective primary isolated on-pump coronary artery bypass grafting (CABG), depending on preoperative continuation of aspirin or its replacement with LMWH, since direct and unbiased comparisons are lacking.

## Method

Retrospectively, 200 patients were included in Group 1, in which aspirin was stopped at least five days before surgery and replaced with enoxaparin, and 200 patients in Group 2, in which aspirin was continued until surgery. Postoperative bleedings and surgical complications were monitored during hospitalization.

## Results

Postoperative bleeding was lower in Group 2 compared to Group 1 in the first hour after operation (p = 0.005), in the following 12 hours from surgery (p < 0.001), and considering the overall blood loss with reduced major postoperative bleeding events rate (p < 0.001). There were not differences in the use of blood products and reoperation for bleeding. Patients in Group 2 tended to have lower values of postoperative C-reactive protein (p = 0.068). Aspirin withdrawal before surgery was an independent predictor of major postoperative bleeding at Logistic regression, while statin treatment might exert a protective effect (p = 0.085). Combined aspirin and statin treatment is even more beneficial (p = 0.031). After propensity score adjustment, aspirin protective effect carries an adjusted odds ratio of 0.317 (p = 0.001).

## Discussion/Conclusion

Postoperative bleeding was reduced in patients who continued aspirin until the day of surgery compared to patients who replaced it with LMWH. This finding may be due to a reduction in postoperative inflammatory reaction, since statin treatment played a protective role and C-reactive protein levels tended to be reduced in patients who continued aspirin.

